# Profile of patients with penile cancer in the region with the highest worldwide incidence

**DOI:** 10.1038/s41598-020-59831-5

**Published:** 2020-02-19

**Authors:** Ciro Bezerra Vieira, Laisson Feitoza, Jaqueline Pinho, Antonio Teixeira-Júnior, Joyce Lages, José Calixto, Ronald Coelho, Leudivan Nogueira, Isabela Cunha, Fernando Soares, Gyl Eanes Barros Silva

**Affiliations:** 1 University Hospital of Federal University of Maranhão (UFMA), Barão de Itapari Street, Centro, São Luís, Brazil; 2 Department of Radiology, University Clinic Hospital of Estadual University of Campinas, Campinas, Brazil; 3 Laboratory of Immunofluorescence and Electron Microscopy (LIME), Presidente Dutra University Hospital (HUUFMA), São Luís, MA Brazil; 4 Postgraduate Program in Adult Health (PPGSAD), Federal University of Maranhão (UFMA), São Luis, MA Brazil; 5 Department of Public Health, Presidente Dutra University Hospital (HUUFMA), São Luís, MA Brazil; 6 Department of Medicine II, Federal University of Maranhão (UFMA), São Luís, MA Brazil; 7 Doctor, Oncologist at the Aldenora Bello Cancer Hospital (HCAB), São Luís, MA Brazil; 8 Doctor, Urologist at the Aldenora Bello Cancer Hospital (HCAB), São Luís, MA Brazil; 9 Doctor at the Antônio Prudente Foundation, São Paulo, Brazil; 10 Department of Pathology, School of Medicine, University of São Paulo, São Paulo, Brazil; 11 Department of Pathology, Ribeirão Preto Medical of School, University of São Paulo (USP), Ribeirão Preto, Brazil

**Keywords:** Cancer epidemiology, Penile cancer

## Abstract

To determine the epidemiological, histopathological, and clinical characteristics of patients diagnosed with penile cancer in the Brazilian state of Maranhão, the region with the highest incidence worldwide. One hundred and sixteen penile cancer patients were interviewed from July 2016 to October 2018. The majority of patients lived in a rural area (57%), worked in farming (58%), had a low level of schooling or no schooling (90%), and were married or in a stable relationship (74%). The mean age was 60.4 ± 16.51 years (range, 23–93 years). Phimosis (66%), poor/moderate genital hygiene (73%), history of sexually transmitted infections (55%), and zoophilia (60%) were found in the majority of patients. Most patients had their first sexual encounter at 16.2 ± 2.8 years (range, 10–25 years), and 75% had >6 sexual partners. The most common initial symptom was pruritus (37%), and most patients waited to seek treatment (average time to treatment, 18.9 months; range, 2–84 months). Human papillomavirus (HPV)-related histologies were observed in 62% of patients. Most patients had histological grades II or III (87%), stage ≥T2 disease (84%), and lymphadenopathy at admission (42%). Penectomy was performed in 96% of patients. The population with penile cancer in the region of highest incidence in the world is marked by low socioeconomic status, high prevalence of HPV infection, and phimosis. The delay in seeking treatment is related to a very high rate of advanced cancer and aggressive surgical treatment. The high prevalence of young patients was also a striking feature.

## Introduction

Penile cancer is uncommon in developed countries, and its incidence varies according to the demographic region and the circumcision status^1,2^. In the US and Europe, the age-standardized incidence rate (ASR) for 100,000 inhabitants is between 0.1 and 1.0^3^. However, in less developed countries, including some countries in South America, Asia, and Africa, the incidence is alarmingly high^1^. In uncircumcised males, the cumulative lifetime risk for cancer of the penis is higher compared to the general population, corresponding to 1 in 600 men in the US^2^. Brazil has a high incidence, and the state of Maranhão, located in the northeastern region of the country, has the highest incidence of penile cancer in the world (ASR of 6.1 cases per 100,000 inhabitants)^4^.

The etiology of penile carcinoma remains unknown, and its mechanisms of development have not been completely elucidated. Phimosis, balanitis, smegma, precarious genital hygiene, smoking, history of sexually transmitted infections (STIs), and human papilloma virus (HPV) infection (present in approximately 50% of cases) are the main risk factors, and circumcision is an important protective factor^5–8^. Its incidence is higher in regions with lower socioeconomic level and schooling, given that the presence of risk factors tends to be higher in these locations^9–11^.

Maranhão is the poorest state in the country, with a per capita income of 155.59 USD and a Human Development Index of 0.639^12^. These factors, together with the high rate of rural inhabitants, distance from major health centers, and little or no schooling, provide the ideal characteristics for the development of penile cancer. Moreover, there is a high HPV prevalence in the general population of the state^13^. Therefore, the objective of this study is to determine the epidemiological, histopathological, and clinical characteristics of patients diagnosed with penile cancer in the region with the highest reported incidence worldwide. Understanding the profile of these patients can help devise strategies to change this reality.

## Methods

A prospective, cross-sectional, descriptive study was performed between July 2016 and October 2018. A total of 116 patients with penile carcinoma treated at the University Hospital of the Federal University of Maranhão and the Aldenora Bello Cancer Hospital (HCAB) in the city of São Luís were included.

The data were collected by a single investigator using a questionnaire. Biopsy samples were analyzed by a uropathologist with 15 years of experience who performed complete paraffin inclusion of the lesion or a minimum of 40 blocks of paraffin for very advanced lesions.

The variables studied included age, marital status, schooling, occupation, residence, family income, smoking, alcoholism, characteristics of the foreskin, history of genital warts, genital hygiene^14^, history of STIs, sexual history, initial symptom and duration of symptoms, lymph node enlargement on admission, tumor size, topography, subtype (World Health Organization, 2018) and histological grade^15^ of the tumor, staging (TNM 8th Ed., 2017), type of surgery, and completion of lymphadenectomy. Information on alcohol intake, history of genital warts, history of STIs, initial symptoms, and zoophilia began to be collected after the study was underway, which explains the low number of cases evaluated. The criterion used to classify genital hygiene as poor/moderate was less than one genital wash with soap per day or presence of phimosis.

Data was arranged using Microsoft Excel 2010 and analyzed with Stata version 12.0 (Stata Corporation, College Station, TX, USA). Statistical analyses were performed using the Chi-square and Fisher’s exact test. The 95% confidence interval was calculated using logistic regression with a significance of p < 0.05. This work was in accordance with the principles of the 1964 Helsinki declaration and approved by the Research Ethics Committee of the Universidade Federal do Maranhão [process no. 43774215.7.0000.5086] and written informed consent was obtained from all patients.

## Results

### Sociodemographic aspects

Between 2016 and 2018, 116 patients were analyzed. Most lived in a rural area (57%), worked in farming (58%), had no schooling or studied only through primary school (90%), and were married or in a stable relationship (74%) (Table 1).

**Table 1:** Sociodemographic profile of patients with penile cancer.

	% (N)
**Marital status**	
Married/stable relationship	74 (72/98)
Widower	13 (13/98)
Not married	12 (12/98)
Divorced	1 (1/98)
**Occupation**	
Farmer	58 (64/110)
Fisherman	6 (7/110)
Others	36 (39/110)
**Schooling**	
No education	37 (37/100)
Primary education	53 (53/100)
Secondary education	10 (10/100)
**Family income**	
<1 minimal wage	82 (77/94)
>1 minimal wage	18 (17/94)
**Residence**	
Rural	57 (64/113)
Urban	35 (40/113)
Suburban	8 (9/113)

### Clinical features

The mean age at diagnosis was 60.4 ± 16.51 years, with a predominance of men over 60 (54%); 22% were younger than 45. There was no relationship between age and advanced tumors (p = 0.65), high histological grade (p = 0.58), or HPV-related subtypes (basaloid, condylomatous, and mixed; p = 0.55). Fewer than half were smokers (41%), more than half had a history of phimosis (66%), and a minority were circumcised (24%). All the circumcised patients underwent the procedure in adulthood after presenting with symptoms. Most men had no genital warts (78%), had poor/moderate genital hygiene (73%), had a history of STIs (55%), and practiced zoophilia (60%). The average age at first sexual encounter was 16.2 years, and the number of lifetime sexual partners was >6 in 75% of patients. The average time from onset of symptoms to seeking treatment was 18. 9 months, and the most common symptom was pruritus (37%). Lymph node enlargement on admission was observed in 42% of the patients (Table 2).

**Table 2:** Clinical features of patients with penile cancer.

	% (N)
**Age [mean (range)]**	**60.4 years (23–93 years)**
Below 40 years	14 (16/116)
Between 40 and 60 years	32 (37/116)
Above 60 years	54 (63/116)
**Smoking habits**	
No	43 (46/107)
Yes	41 (44/107)
Not evaluated	16 (17/107)
**Alcoholism**	
No	30 (29/96)
Yes	24 (23/96)
Former alcoholist	9 (9/96)
Not evaluated	37 (35/96)
**Foreskin characteristics**	
Long foreskin	16 (16/98)
Phimosis	66 (71/110)
Previous circumcision	24 (26/110)
**Genital warts**	22 (14/65)
**Genital hygiene**	
Poor/moderate	73 (70/96)
Good	27 (26/96)
**History of STIs**	55 (40/73)
**Sexual history**	
Age of first sexual encounter [mean (range)]	16.2 years (10–25 years)
Zoophilia	60 (43/72)
**No. female partners**	
Less than 6	25 (19/75)
Between 6 and 10	17 (13/75)
More than 10	58 (43/75)
**Duration of symptom [mean (range)]**	18.9 months (2–84 months)
**Initial symptom**	
Prutirus	37 (24/64)
Ulcer	28 (18/64)
Nodule/tumor	26 (17/64)
Secretion	11 (7/64)
Others*	11 (7/64)
**Linfo node enlargement on admission**	42 (28/67)

*Others: pain, dyspareunia, dysuria, oliguria, swelling and inflammation.

### Histopathological and surgical aspects

All patients were diagnosed with squamous cell carcinoma. The average tumor size was 4.5 cm (largest diameter), with the most frequent location being the glans (47%). The usual subtype was that most commonly found in the samples (40%), but HPV-related types were more representative when considered together (62%). HPV subtype was not related to high histological grade (p = 0.16) or advanced tumor stage (p = 0.63). There was a predominance of histological grades II and III (87%). Partial penectomy was the most common surgical treatment (64%), and lymphadenectomy was performed in 52% of the patients (Table 3).

**Table 3:** Pathological aspects, staging and treatment of patients with penile cancer.

	% (N)
**Tumor size [mean (variation)]**	4.50 cm (0.7–10 cm)
**Tumor topography**	
Glans	47 (52/112)
Glans and foreskin	25 (28/112)
Glans and corpus	10 (11/112)
Foreskin	3 (3/112)
Foreskin and corpus	1 (1/112)
Glans, foreskin and corpus	14 (16/112)
**Histological subtypes**	
Usual	40 (44/110)
Warty	33 (36/110)
Basaloid	5 (5/110)
Mixed*	25 (27/110)
Others**	5 (6/110)
**Histological grade**	
Grade I	13 (14/111)
Grade II	46 (51/111)
Grade III	41 (46/111)
**Primary tumor (T)**	
Tis	2 (2/111)
T1(a,b)	14 (16/111)
T2	29 (32/111)
T3	46 (51/111)
T4	7 (8/111)
Tx	2 (2/111)
**Type of surgery**	
Parcial penectomy	64 (74/116)
Total penectomy	26 (30/116)
Emasculation	7 (8/116)
Others (glansectomy, postectomy and excision)	3 (4/116)
**Lymphadenectomy**	
Unilateral	20 (17/86)
Bilateral	32 (28/86)
Not performed	48 (41/86)

*Mixed: 18 usual and warty, 4 usual and basaloid, 1 warty and basaloid, 1 warty and acantholitic.

**Others: papillary.

## Discussion

Brazil has one of the highest incidences of penile cancer worldwide. This incidence is much higher than in the United States and Europe (0.4–0.6%)^8,16^, only resembling developing nations like India, African and others South American nations^1,17^. However, there is variability even within Brazil, where the highest number of cases is reported in the Northeast region^18^. The incidence in this region is 5.7%, surpassing the economically more developed region - Southeast (1.2%) - and the North (3.8%), the second poorest region^19^. The particularly high number of patients with penile cancer in Maranhão, which has the highest reported incidence worldwide, accounts for this state being the least developed region of Brazil and having high rates of HPV infection^12,13^.

The sociodemographic profile in this study were very similar to those of other localities in Brazil and worldwide^10,18–20^. The patients were mainly of low economic status and education and lived rurally. However, unlike most studies, there were significant numbers of patients living in urban and suburban areas. A similar profile was only found in the state of Pará^21^ and in Paraguay^14^, localities with similar sociodemographic aspects. This is probably due to the recent demographic transition that occurred in these regions, with migration of rural inhabitants to large centers in search of better living standards, employment, and healthcare. Despite living in developed regions, these patients have a similar socioeconomic profile to those in rural areas, leaving them vulnerable to the same risk factors for the development of neoplasia^14^.

Penile cancer is more common in older men^1^. However, it can occur in young men, and the incidence in men <45 years in Brazil is high (19.41%)^18^. Our data followed this trend, with 22% men being in this age range. There are few published studies on penile cancer in the young population, so it is unclear whether age is related to increased tumor aggressiveness^22^. In our sample, there was no relationship between age and advanced tumor stage, high histological grade, or HPV subtype. Nevertheless, our numbers are worrisome, since mutilating treatments, like emasculation and partial or total penectomy, have been instituted in sexually active men, subjecting them to physical sequelae, negative impact on welfare, psychological and sexual dysfunction^23,24^. We also saw a concerning delay in seeking treatment. The average interval from the onset of symptoms to first treatment was 18.9 months. Patients with penile cancer have the longest delay in seeking treatment, reaching more than 1 year in 15–50% of cases and the most common causes for this are fear, embarrassment, and social stigma^17^. Further, because Maranhão is the poorest state in the country, there is a serious structural and healthcare deficit, with difficulty in accessing adequate treatment. The result is a high number of men with advanced neoplasms and mutilating treatments.

A minority of patients in our sample were smokers (41%). Tobacco is an established risk factor for penile cancer and other neoplasms^20,25,26^. Hellberg *et al*.^25^ also noted that heavy smokers have a higher risk for penile carcinoma. Compared to other localities, Maranhão has the smallest proportion of smokers among patients with penile cancer (Rio de Janeiro^10^: 56.5%, Canada^27^: 50%, and Paraguay^14^: 76%). This indicates that penile cancer in Maranhão is more strongly related to other risk factors.

Phimosis, inflammatory conditions (balanitis and lichen sclerosus) and poor genital hygiene are relevant risk factors for cancer of the penis. The prevalence of phimosis in males in adulthood is over 3.4%^28^. Between patients with cancer of the penis, this prevalence is over 25–75%^16,29^, and its contribution can be attributed to the accumulation of smegma, higher incidence of HPV or as an isolated carcinogenic factor^7,30–32^. Genital lichen sclerosus was associated with malignant penile changes in 5.8%^33^ of cases and balanitis over 45%^11^. Conversely, circumcision, if performed in the neonatal period or up to adolescence, is an important protective factor^34^. In uncircumcised individuals, good genital hygiene is perhaps the most important protective measure^6,35^. In our study, the prevalence of phimosis was high, and the majority of circumcised men underwent the procedure in adulthood, after having symptoms of the disease. This profile is similar to the national tendency^18^: the prevalence of phimosis among men with penile cancer in Brazil is 60%, and only 13% of patients are circumcised. This is because of the low prevalence of circumcision in the general population (<20%)^36^. Neonatal circumcision is not normally performed in Brazil, and regions with worse access to healthcare have a lower rate of circumcision in childhood and adulthood^37^. By comparison, in the United States, where around 80.5% of the global population is circumcised, the incidence of penile cancer is very low^38^.

Factors related to sexual history are of fundamental importance in the development of penile cancer. HPV is present in 15–80% of cases, and condylomatous, basaloid, and mixed (with condylomatous and/or basaloid component) histologies are related to HPV infection^39,40^. The pivotal multinational study conducted by Castellsague *et al*.^41^ stratified the susceptible to acquiring HPV infection in men according to sexual behavior in high risk (individuals who had ≥6 sexual partners and a sexual debut before the age of 17), low risk (individuals reporting ≤5 partners and the first sexual experience at ≥17 years of age), and medium risk (other than low or high risk individuals). In our study, the prevalence of HPV-related subtypes was 60%; in men with 6 or more sexual partners, it was 75%, and in men who had their first sexual intercourse before the age of 17 was 59%. This is due to the very high prevalence of HPV in Maranhão; the state capital has the highest prevalence in Brazil (59.1%)^13^. Early onset of sexual activity and a large number of sexual partners contribute to this high prevalence.

The relationship between zoophilia and penile cancer was the subject of a recent study^42^. The authors consider zoophilia a risk factor and demonstrate that men who practiced sex with animals are two times more likely to develop cancer of the penis. They believe that microtraumas generated in the penis and contact with animal secretions are the causative factors. In our study, 60% of patients practiced zoophilia.

The signs and symptoms of penile cancer are varied. The most frequent initial sign is a change in the skin of the penis, such as nodules, ulcers, swelling, and color changes^34^. A few studies include pruritus as an initial symptom, and, when present, it is more commonly related to associated skin lesions, such as lichen sclerosus^33,43^. Interestingly, in our study, intense pruritus was the most common initial symptom and was described as the only initial symptom in 26.56% of patients. However, the presence of lichen sclerosus was extremely low (6%), which indicates the possibility of other causes in our sample that can explain pruritus.

The number of patients with characteristics of advanced disease, like lymph node enlargement on admission (41.8%), high histological grade (87%), and T stage ≥2 (82%) was high. These are higher than those in other Brazilian studies^10,20^ and studies in developed nations such as Finland^44^ and the USA^45^. Although histological subtypes related to HPV represented the major part of the sample, there was no significant relationship with high grade (p = 0.16) or advanced stage (p = 0.63). This can be explained by the less aggressive natural behavior of HPV-related tumors^46,47^.

Penectomy was performed in almost all cases, surpassing the proportion of patients undergoing radical surgery in other Brazilian regions (80.9%–93.1%)^10,18^, the United States (82.1%)^45^ and Canada (71%)^27^. The high proportion of penectomies over less invasive surgical alternatives, such as local tumor excision, is due to the large number of advanced tumors in our sample. Although penectomy is the gold standard therapy for penile cancer, its physical and psychosocial effects are devastating^43^. The number of lymphadenectomies was also high, irrespective of lymph node metastasis, because lymphadenectomy was performed prophylactically in some patients due to the difficulty in maintaining follow-up.

One limitation of this study is that it does not cover all hospitals in the state, and therefore may underestimate the actual number of cases. In addition, the low educational and socioeconomic level of some patients hampered the collection of reliable data in some cases, although these were excluded from the analysis. However, it was possible to determine important factors associated with cancer of the penis in a region of high incidence.

## Conclusion

The population with penile cancer in the region with the highest worldwide incidence is marked by a high prevalence of HPV infection and phimosis. Together with the low socioeconomic level of the patients, these are leading factors for the development of the neoplasm. A delay in seeking treatment was related to the high frequency of advanced tumors and aggressive surgical treatment. The high prevalence of young patients was also a striking feature. The reduction through vaccination against HPV, early infant circumcision, hygiene measures, early diagnosis, and education can stop the advance of the neoplasm in the state.

## Data Availability

The datasets used and/or analyzed during the current study are available from the corresponding author on reasonable request.
